# Psychological assessment of alcoholism in males

**DOI:** 10.4103/0019-5545.31602

**Published:** 2006

**Authors:** Suprakash Chaudhury, S.K. Das, B. Ukil

**Affiliations:** * Professor and Head Department of Psychiatry, RINPAS; ** Consultant Psychiatrist Bhubaneshwar; *** Psychiatrist No. 3 CRPF Base Hospital, Guwahati

**Keywords:** Alcohol dependence, alexithymia, self-esteem, anxiety, depression, life events

## Abstract

**Background:**

Little work has been done in India on the personality factors of alcoholics. These personality factors have a significant effect on treatment outcome.

**Aim:**

To study the personality characteristics, stressful life events and diagnostic utility of the Michigan Alcoholism Screening Test (MAST) and CAGE (Cutting down, Annoyance by criticism, Guilty feeling, and Eye-opener) Questionnaire in service personnel with alcohol dependence.

**Methods:**

Psychological assessment of 100 consecutive male inpatients meeting the DSM-IV criteria for alcohol dependence, and an equal number of controls matched for age, sex, occupation and regional background was carried out utilizing the MAST, CAGE Questionnaire, State-Trait Anxiety Inventory, Hamilton Rating Scale for Depression, Multiphasic Personality Questionnaire, Maudsley Personality Inventory, Toronto Alexithymia Scale, Self-esteem Inventory and Presumptive Stressful Life Events scale.

**Results:**

The MAST and CAGE were of limited value in the diagnosis of alcohol dependence. Alcoholics obtained significantly higher scores on state and trait anxiety, depression, mania scale, paranoia scale, schizophrenia scale, psychopathic deviance, neuroticism, extroversion, and the Presumptive Stressful Life Events scale. Alcohol-dependent individuals had significantly lower self-esteem compared with control subjects, and significantly more alcoholics were identified as alexithymic.

**Conclusion:**

Alcohol-dependent individuals show significantly high neuroticism, extroversion, anxiety, depression, psychopathic deviation, stressful life events and significantly low self-esteem as compared with normal control subjects. Significantly more alcoholics were found to be alexithymic compared with normal controls.

## INTRODUCTION

Concerted attempts have been made to relate personality factors to alcohol dependence. Potential alcoholics tend to be emotionally immature, expect a great deal of the world, require an inordinate amount of praise and appreciation, react to failure with marked feelings of hurt and inferiority, have a low frustration tolerance, and feel inadequate and unsure of their abilities to fulfil expected male or female roles.^1^ Certainly, many people with similar personality characteristics do not become alcoholics, and others with dissimilar ones do. One characteristic that appears common to the backgrounds of most problem drinkers is personal maladjustment, yet most maladjusted people do not become alcoholics. Although the significance of alcoholic personality factors remains unclear, researchers have shown that the personality of alcoholics significantly influences treatment outcome.^2^ Hence, an understanding of the personality characteristics associated with alcohol dependence may be useful for treatment.

Early diagnosis is another factor that influences treatment. Often, short questionnaires such as the MAST (Michigan Alcoholism Sreening Test) and CAGE (Cutting down, Annoyance by criticism, Guilty feeling, and Eye-opener) are used to screen populations for alcohol dependence. Researchers have long been interested in how individuals and environments affect each other, primarily to be able to explain age-related behaviour and individual differences. One focus has been to study life events. Through the life events scale we can get a simple index of life stress.

Comparatively little work has been done in this field in India and no work has been carried out among the security forces, where alcohol dependence is a major problem.^3^ This prompted us to focus on a few psychological tests in an effort to reveal the personality characteristics and stressful life events in alcohol-dependent individuals, and to study the diagnostic utility of the MAST and CAGE among service personnel.

## METHODS

One hundred consecutive male inpatients admitted to the psychiatric centres of two Base Hospitals and meeting the DSM-IV criteria^4^ for alcohol dependence were included in the study with their informed consent. An equal number of subjects matched for age, sex and regional background without any physical or psychiatric illness formed the control group. Exclusion and inclusion criteria have been given elsewhere.^5^

The mean age (± SD) of the patients and control subjects was 38.05 (± 7.36) years and 38.57 (± 7.32) years, respectively (range 25–54 years for both groups). There were no significant differences between the two groups on sociodemographic variables such as age, service, rank, marital status, education, religion and domicile. All the subjects were male. All the subjects were administered the following psychological tests after 7–9 days of hospitalization:

1. MAST^6^
2. CAGE Questionnaire^6^
  Both these screening instruments were used to compare their diagnostic utility in service personnel.
3. State-Trait Anxiety Inventory (STAI)^6^—measures anxiety: At present (state) and habitual (trait)
4. Hamilton Rating Scale for Depression (HRSD)^6^
5. Toronto Alexithymia Scale (TAS)^6^
6. Maudsley Personality Inventory (MPI)^7^—to assess neuroticism and extroversion
7. Multiphasic Personality Questionnaire (MPQ)^8^,^9^—to assess the presence of paranoid, hysteria, schizophrenic, anxiety, mania, depression and psychopathic features
8. Self-Esteem Inventory (SEI)^10^
9. Presumptive Stressful Life Events (PSLE) scale^11^

The control subjects also underwent all the tests given to the patients. Statistical analysis was carried out using the Mann–Whitney U test and Chi-square test with Yates correction as appropriate.

## RESULTS

On the MAST, 69 patients and 6 control subjects scored in the alcoholic range, the difference being highly statistically significant (χ^2^=84.7; df=1; p<0.01). On the CAGE, 66 patients and 5 control subjects scored in the alcoholic range. The difference was highly statistically significant (χ^2^=78.61; df=1; p<0.01). Sensitivity, specificity and predictive values of the MAST and CAGE for alcohol dependence are given in Table 1.

**Table 1:** Sensitivity, specificity and predictive values for alcohol dependence of the MAST and the CAGE questionnaire

Test	MAST	CAGE
Sensitivity	69	66
Specificity	94	95
False-positivity rate	6	5
False-negativity rate	41	44
Positive predictive power	92	92.5
Negative predictive power	69.6	68.3
Overall diagnostic power	81.5	80.5

All values are percentages

The results of the other psychological tests are given in Table 2. Alcohol-dependent individuals obtained significantly higher scores on neuroticism and extroversion on the MPI.

**Table 2:** Psychological test results of alcohol-dependent patients (*n*=100) and matched control subjects (*n*=100)

Psychological tests	Alcohol-dependent patients Mean (SD)	Normal control subjects Mean (SD)
Maudsley Personality Inventory		
Neuroticism	39.2 (6.4)*	30.2 (4.1)
Extroversion	50.9 (10.6)*	41.2 (5.4)
Multiphasic Personality Questionnaire		
Paranoia scale	6.1 (2.48)*	5.2 (1.61)
Hysteria scale	1.4 (1.11)	2.1 (1.31)
Mania scale	5.7 (1.50)*	4.6 (1.43)
Depression scale	5.1 (1.99)*	3.5 (1.29)
Schizophrenia scale	3.8 (1.85)*	2.7 (1.52)
K scale	4.3 (1.28)	4.1 (1.39)
Pd scale	15.9 (2.14)*	8.5 (1.31)
Anxiety scale	9.6 (4.39)*	5.2 (1.68)
State-Trait Anxiety Inventory		
State anxiety	51.3 (1.3)*	37.8 (9.5)
Trait anxiety	48.6 (10.7)*	36.3 (8.3)
Hamilton Rating Scale for Depression	9.37 (3.9)*	1.1 (1.4)
Toronto Alexithymia Scale	64.4 (20.9)	59.5 (17.3)
Score >74	26*	12
Presumptive Stressful Life		
Events scale		
One year	3.7 (1.45)*	1.9 (1.36)
Lifetime	15.7 (3.64)*	12.1 (3.47)
Self-Esteem Inventory	16.37 (3.1)*	12.03 (2.7)

* Significantly different from controls

On the MPQ, alcohol-dependent patients scored significantly higher on paranoia, mania, depression, schizophrenia, psychopathic deviance (Pd) and the anxiety scale. Compared to control subjects, alcohol-dependent patients had significantly higher scores on state and trait anxiety, depression and stressful life events. On the Self-Esteem Inventory, alcohol-dependent patients scored significantly higher, indicating markedly lower self-esteem among them compared with normal subjects. There was no statistically significant difference on the TAS scores between alcohol-dependent and control subjects. However, a TAS score greater than 74 is recommended as the cut-off point for identifying alexithymic people. Based on this, 26 alcoholics and 12 normal subjects were identified as alexithymic, the difference being highly statistically significant (χ^2^=7.11; df=1; p<0.01).

## DISCUSSION

In the present study, with the DSM-IV diagnosis as the standard, the MAST and CAGE Questionnaire showed a high specificity (about 94%) and high positive predictive value (about 92%), but their sensitivity for the DSM-IV diagnosis of alcohol dependence was rather low (69% and 66%, respectively). Thus, the MAST and CAGE are of limited utility as screening instruments; in future studies in security forces the CAGE may be preferred because of its brevity. In support of this, it has been reported that the positive predictive value of the MAST in unselected clinical samples as well as in epidemiological surveys is rather low.^12^ It is noteworthy that the MAST and CAGE only assess the lifetime risk of severe alcohol problems and so may have a lower sensitivity for current alcohol dependence.

The finding of significantly higher scores on extroversion indicate that alcohol-dependent subjects are characterized by traits such as being more assertive, dominant, sociable, carefree and venturesome as compared to non-dependent people. This finding is in agreement with that of Mathew and Baby^13^ but not of King *et al.*^14^ Alcohol-dependent patients also obtained significantly higher scores on the neuroticism dimension. This indicates that they are significantly more emotional, frequently anxious and/or depressed, moody and tense. Similar results were reported in earlier studies.^12^,^15^

Among the personality traits studied in alcohol-dependent individuals, antisocial personality has been looked into most often.^1^ In the present study, alcohol-dependent subjects obtained significantly higher scores on the Pd scale of the MPQ, which is in agreement with the findings of Neeliyara *et al.*^16^ A longitudinal study of men older than 40 years also revealed that antisocial behaviour in adolescence is the sole individual predictor of alcoholism.^17^ However, it must be pointed out here that the high Pd scores in alcohol-dependent patients indicate a transitory state, which may be amenable to change with treatment. Our finding that alcohol-dependent patients showed disturbances in the depression, mania, schizophrenia, psychopathic deviance and anxiety scales is consistent with previous research that the disturbance in the MPQ in people with substance abuse is broad-based, variable and non-specific.^18^

Alcohol-dependent individuals also obtained significantly higher trait and state anxiety scores (Table 2). These findings support those of a few earlier studies.^14^,^16^ This aspect may be aetiologically significant in alcohol dependence. Anxiety has been suggested to be an important factor in the initial development and subsequent maintenance of alcohol abuse and dependence. Some patients use alcohol as a medication for the treatment of anxiety. Unfortunately, an accurate diagnosis of anxiety disorders is difficult to make, since current anxiety symptoms may be secondary to alcohol withdrawal rather than reflecting underlying anxiety disorders.^19^ The findings of the present study also reveal that alcohol-dependent individuals are different from those with anxiety neurosis, since they have a high state anxiety unlike those with anxiety neurosis, who have a high trait anxiety. This indicates that anxiety in alcohol-dependent individuals is transitory, varies in intensity and fluctuates over time, and can be easily modified. One of the sources of anxiety is a low level of self-esteem, fear of disapproval from significant people, loss of position, prestige, stature or self-esteem.^16^ Thus, these findings also support our finding that alcoholics have low self-esteem, which underlies the need for reducing anxiety using suitable therapeutic interventions.

The finding of significantly higher depression scores in alcohol-dependent persons (Table 2) is in agreement with earlier work.^14^,^15^ While some patients may use alcohol as ‘self-medication’ for their depression, alcohol itself may produce clinically significant depression. Clinicians obviously need to carefully assess alcohol-dependent individuals for depression, which must also be treated.

Patients with alcohol dependence experience significantly more stressful life events in the past year and over their lifetime (Table 2). These findings are in line with previous reports.^13^,^14^,^20^ Men who are lifelong abstainers experience fewer life events than problem drinkers; in turn, problem drinkers experience less stress than alcoholics.^14^ Alcoholics may offset stress-induced emotional distress by resorting to drink which, in turn, might lead to a further increase in negative life events.

A person's self-structure is an important aspect of his personality. A healthy personality is manifested when an individual has a positive attitude towards him/herself. Studies in this area have shown that psychiatric patients have unhealthy self-structures by way of poor self-concept. In this view, alcohol-dependent individuals suffer from lowered feelings of self-esteem, pervasive feelings of inferiority and powerlessness, coupled with unusually strong inhibitions against the expression of hostile or aggressive impulses. In the present study, alcohol-dependent individuals had significantly lower self-esteem as compared with normal subjects. This finding is in agreement with that of Neeliyara *et al.*^16^ This indicates that alcohol-dependent individuals have less positive self-feelings and more feelings of alienation and isolation.

Though alcohol-dependent patients and normal subjects in the present study did not differ in the mean alexithymia scores (Table 2), significantly higher numbers of alcohol-dependent subjects were identified to have alexithymia. This finding is congruent with earlier work.^15^,^21^ It has been reported that in recently sober alcoholics the alexithymic cognitive dimension—an inability to identify feelings and to distinguish them from bodily sensations—is related to depressive symptoms and suicidal ideation. The addictive behaviour of these people signifies desperate attempts at self-treatment.^15^,^21^

From the present study, it can be concluded that alcohol-dependent individuals show significantly high neuroticism, extroversion, anxiety, depression, psychopathic deviation and significantly low self-esteem as compared to normal control subjects. Significantly more alcoholics were found to be alexithymic. Future studies should assess whether identification of these personality characteristics and institution of suitable psychosocial interventions can improve the outcome of therapy for the alcohol-dependence syndrome in security personnel.
